# Paroxysmal Nocturnal Hemoglobinuria-Driven Hepatic Vein Thrombosis: A Case of Budd-Chiari Syndrome in Disguise

**DOI:** 10.7759/cureus.97611

**Published:** 2025-11-23

**Authors:** Shaily Agrawal, Sai Prasad, Jay Nagda, Vidhi Parmar, Advaitha Reddy

**Affiliations:** 1 Internal Medicine, Shri MP Shah Government Medical College, Jamnagar, IND; 2 Internal Medicine, S Nijalingappa Medical College, Bagalkot, IND; 3 Internal Medicine, Narendra Modi Medical College, Ahmedabad, IND; 4 Community Medicine, Shri MP Shah Government Medical College, Jamnagar, IND; 5 Internal Medicine, Dr. D. Y. Patil Medical College, Hospital & Research Centre, Mumbai, IND

**Keywords:** budd–chiari syndrome, complement inhibition, eculizumab, hepatic vein thrombosis, intravascular hemolysis, paroxysmal nocturnal hemoglobinuria

## Abstract

Paroxysmal nocturnal hemoglobinuria (PNH) is a rare acquired hematopoietic stem cell disorder characterized by complement-mediated intravascular hemolysis and a markedly increased risk of thrombosis, including at atypical splanchnic sites. We report a 26-year-old woman who presented with progressive abdominal distension, right upper quadrant pain, early satiety, and morning dark-colored urine. Investigations revealed normocytic anemia, biochemical evidence of intravascular hemolysis, moderate ascites, hepatomegaly, and elevated D-dimer; direct and indirect antiglobulin tests were negative. Flow cytometry using FLAER (Fluorescent AERolysin) and CD55/CD59 confirmed large PNH clones in granulocytes and monocytes, consistent with classic PNH complicated by hepatic vein thrombosis, Budd-Chiari syndrome (BCS). She received therapeutic anticoagulation and complement inhibition with eculizumab, alongside supportive transfusions and monitoring, with progressive clinical and laboratory improvement. This case emphasizes that BCS may be the presenting manifestation of PNH in young patients without conventional thrombotic risk factors and highlights the importance of prompt flow cytometry testing and initiation of complement inhibition to reduce morbidity and recurrence of thrombotic events.

## Introduction

Paroxysmal nocturnal hemoglobinuria (PNH) is an acquired, non-malignant clonal hematopoietic stem cell disorder caused by somatic mutations in the *PIGA* gene. These mutations disrupt glycosylphosphatidylinositol (GPI) anchor biosynthesis, resulting in a deficiency of protective surface proteins such as CD55 and CD59. Their absence renders red blood cells susceptible to complement-mediated destruction, leading to chronic intravascular hemolysis. Clinically, PNH is characterized by a triad of hemolysis, bone marrow failure, and thrombosis, the latter being the major cause of mortality. Thrombosis commonly occurs at atypical venous sites such as the hepatic, portal, and cerebral veins. Budd-Chiari syndrome (BCS), defined by hepatic vein thrombosis, is a relatively rare but classical and potentially fatal manifestation of PNH. Based on the acuity of symptom progression, the complication was categorized as subacute. Early recognition in young patients without conventional thrombotic risk factors is crucial. Flow cytometry using FLAER (Fluorescent AERolysin) and CD55/CD59 markers remains the diagnostic gold standard and guides timely therapy with complement inhibitors.

## Case presentation

A 26-year-old female software engineer presented with dull aching right upper quadrant pain and progressive abdominal distension for four weeks. She reported dark-colored urine, especially in the mornings, generalized fatigue, and intermittent mild icterus for approximately three to four months. There was no history of alcohol use, oral contraceptive use, chronic liver disease, gastrointestinal bleeding, or prior thrombotic disorders. A urine pregnancy test was negative.

On admission, the patient’s vital signs were stable: blood pressure 110/70 mmHg, pulse 88/min, temperature 98.6°F, and respiratory rate 18/min. She appeared mildly icteric, with moderate ascites and tender hepatomegaly palpable 4 cm below the right costal margin. There were no signs of chronic liver disease, pedal edema, or splenomegaly. Cardiovascular and respiratory system examinations were unremarkable.

Initial laboratory investigations revealed anemia, thrombocytopenia, and evidence of hemolysis. Liver function tests showed a mixed hepatocellular-cholestatic pattern, and coagulation studies indicated mild prolongation of prothrombin time and elevated D-dimer, suggesting a prothrombotic state. The detailed results of laboratory parameters are summarized in Table 1.

**Table 1:** Laboratory Investigations With Reference Ranges Laboratory profile of the patient demonstrating anemia, thrombocytopenia, indirect hyperbilirubinemia, elevated LDH, and markedly reduced haptoglobin, consistent with intravascular hemolysis. Elevated D-dimer and prolonged PT indicate a prothrombotic state. Flow cytometry confirmed a deficiency of CD55 and CD59, diagnostic of paroxysmal nocturnal hemoglobinuria (PNH). *Reference (normal) ranges were taken from refs. [1-3]. CBC: complete blood count, RBC: red blood cell, MCV: mean corpuscular volume, MCH: mean corpuscular haemoglobin, MCHC: mean corpuscular haemoglobin concentration, WBC: white blood cell, LFTs: liver function tests, AST: aspartate aminotransferase, SGOT: serum glutamic-oxaloacetic transaminase, ALT: alanine aminotransferase, SGPT: serum glutamic-pyruvic transaminase, RFTs: renal function tests, HPF: high-power field, PT: prothrombin time, INR: international normalised ratio, aPTT: activated partial thromboplastin time, FEU: fibrinogen-equivalent units, LDH: lactate dehydrogenase, PNH: paroxysmal nocturnal haemoglobinuria, HBsAg: hepatitis B surface antigen, HCV: hepatitis C virus.

Parameter	Patient Value	Reference Range*	Interpretation
Complete blood count (CBC)			
Hemoglobin	9.8 g/dL	12.0–15.5 g/dL	↓ (Anemia)
RBC count	3.2 × 10⁶/µL	3.8–5.2 × 10⁶/µL	↓
Hematocrit	30 %	36–46 %	↓
MCV	93 fL	80–96 fL	Normal
MCH	30 pg	27–33 pg	Normal
MCHC	32 g/dL	32–36 g/dL	Normal
WBC count	4.2 × 10³/µL	4.0–10.0 × 10³/µL	Normal
Platelet count	95 × 10³/µL	150–400 × 10³/µL	↓ (Thrombocytopenia)
Reticulocyte count	5.5 %	0.5–2.0 %	↑ (Hemolysis)
Liver function tests (LFTs)			
Total bilirubin	3.6 mg/dL	0.2–1.2 mg/dL	↑
Direct bilirubin	0.6 mg/dL	<0.3 mg/dL	↑
Indirect bilirubin	3.0 mg/dL	0.2–0.9 mg/dL	↑ (Unconjugated)
AST (SGOT)	86 U/L	10–40 U/L	↑
ALT (SGPT)	64 U/L	7–56 U/L	↑
Alkaline phosphatase	245 U/L	40–130 U/L	↑
Albumin	3.4 g/dL	3.5–5.0 g/dL	Slightly ↓
Total protein	6.2 g/dL	6.0–8.3 g/dL	Normal
Renal function tests (RFTs)			
Blood urea	28 mg/dL	15–40 mg/dL	Normal
Serum creatinine	0.9 mg/dL	0.6–1.1 mg/dL	Normal
Urine analysis			
Color	Dark/tea-colored	Pale yellow	Abnormal
Protein	Trace	Nil	+
Hemoglobin	Positive	Negative	+ (Hemoglobinuria)
RBCs	Nil	0–3/HPF	Normal
Coagulation profile			
Prothrombin time (PT)	18.2 sec	11–15 sec	↑
INR	1.6	0.8–1.2	↑
aPTT	35 sec	25–35 sec	Normal
Fibrinogen	320 mg/dL	200–400 mg/dL	Normal
D-dimer	1.8 µg/mL FEU	<0.5 µg/mL FEU	↑ (Thrombosis)
Hemolysis markers			
LDH	785 U/L	140–280 U/L	↑↑
Haptoglobin	<10 mg/dL	30–200 mg/dL	↓ (Consumed in hemolysis)
Flow cytometry (PNH panel)			
CD55 deficiency	Present	Absent	Positive for PNH clone
CD59 deficiency	Present	Absent	Positive for PNH clone
Serological tests			
ANA	Negative	Negative	Normal
Viral hepatitis (HBsAg, HCV)	Negative	Negative	Normal

The peripheral smear revealed normocytic, normochromic red cells with a few fragmented forms (schistocytes), supporting intravascular hemolysis. The combination of elevated LDH, increased reticulocyte count, low haptoglobin, and unconjugated hyperbilirubinemia was diagnostic of ongoing hemolysis. Both direct and indirect Coombs tests were negative, excluding autoimmune hemolytic anemia.

Flow cytometry demonstrated 53% Type III granulocytes, 49% Type III monocytes, and 12% CD55⁻/CD59⁻ erythrocytes. The lower percentage of deficient erythrocytes reflects selective complement-mediated destruction of GPI-deficient RBCs, whereas granulocyte and monocyte clone sizes better represent the true PNH clone burden. In PNH, Type I cells show normal GPI-anchored protein expression, Type II cells show partial deficiency, and Type III cells exhibit complete deficiency; high Type III proportions, as seen in this case, are characteristic of classical PNH.

Ultrasonography revealed hepatomegaly (17.2 cm) and moderate ascites. Doppler ultrasonography demonstrated absent flow in the right and middle hepatic veins with preserved portal vein patency, confirming hepatic venous outflow obstruction consistent with BCS. High-resolution images are included, marking the ascitic pockets and non-visualization of hepatic veins.

**Figure 1:**
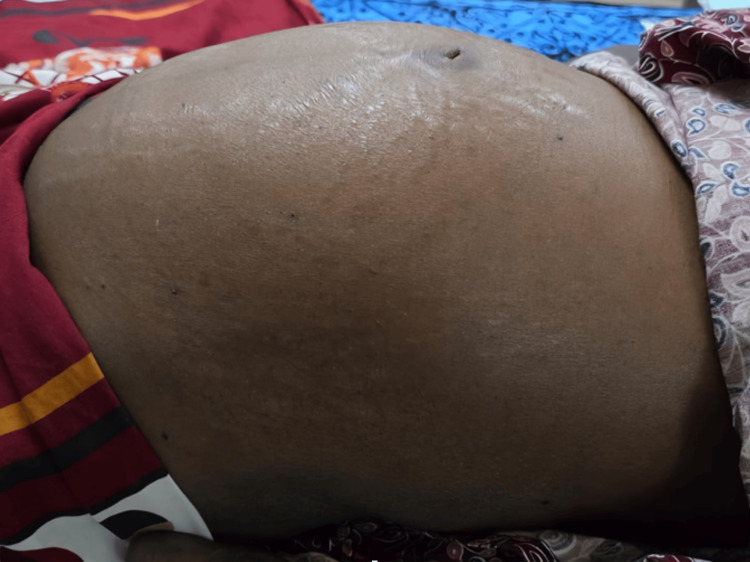
Abdominal Distension in PNH With Budd-Chiari Syndrome Tense abdominal distension with striae in a patient with paroxysmal nocturnal hemoglobinuria (PNH) and Budd-Chiari syndrome, indicating significant ascites due to hepatic outflow obstruction.

**Figure 2:**
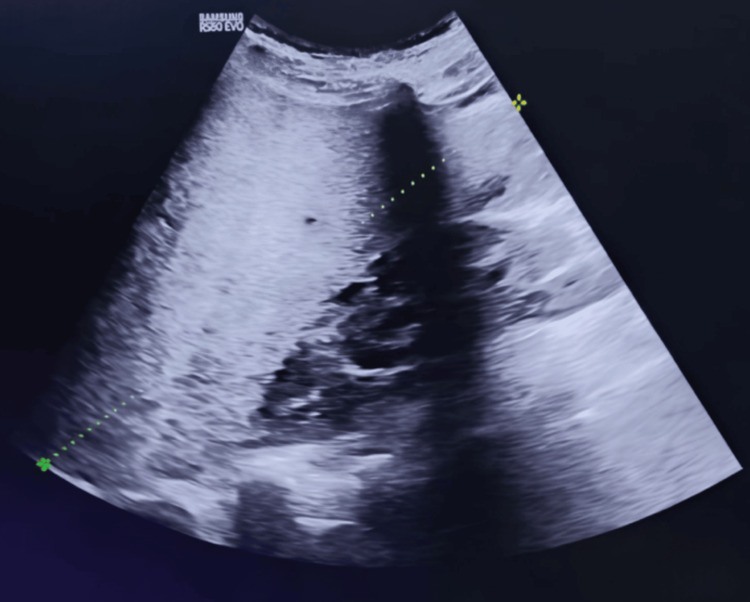
Ultrasound Showing Gross Ascites Abdominal ultrasound revealing anechoic fluid collection consistent with gross ascites, supporting the diagnosis of Budd-Chiari Syndrome in a patient with paroxysmal nocturnal hemoglobinuria.

Clinical inspection revealed tense abdominal distension with visible striae, indicative of significant ascites (Figure 1). Ultrasonography confirmed the presence of gross anechoic ascitic fluid, establishing the diagnosis of BCS in the context of PNH (Figure 2).

Diagnosis

A final diagnosis of PNH with BCS secondary to hepatic vein thrombosis was established based on clinical presentation, biochemical evidence of intravascular hemolysis, and flow cytometric confirmation of GPI-anchor protein deficiency. Differential diagnoses, including alcoholic hepatitis, viral hepatitis, autoimmune hepatitis, and right-sided heart failure, were considered but excluded through history, serological testing, and imaging findings.

Management and follow-up

The patient received meningococcal conjugate vaccination two weeks prior to complement inhibition therapy. Eculizumab was initiated at 600 mg IV weekly for four weeks, followed by a 900 mg loading dose in week five, then 900 mg every two weeks for maintenance. Therapeutic anticoagulation with enoxaparin 1 mg/kg every 12 hours was transitioned to warfarin, targeting an INR of 2-3. Indefinite anticoagulation was advised due to persistent thrombotic risk.

Supportive therapy included transfusion of two units of leukoreduced, irradiated packed RBCs. Weekly monitoring of hemoglobin, LDH, bilirubin, and reticulocyte count during induction and biweekly monitoring during maintenance showed progressive improvement.

At one-month follow-up, hemoglobin improved to 11.2 g/dL, LDH decreased to 290 U/L, and total bilirubin normalized to 1.4 mg/dL. Ultrasound showed reduced ascites. At three months, the patient remained asymptomatic with a stable clone size. Total follow-up duration was four months.

## Discussion

PNH is a rare, acquired clonal hematopoietic stem cell disorder characterized by complement-mediated intravascular hemolysis, bone marrow failure, and an exceptionally high risk of thrombosis, particularly in atypical venous sites such as the hepatic veins, leading to BCS [4]. Thrombosis is the leading cause of mortality in PNH, and hepatic vein thrombosis is a classical but uncommon presentation that often poses significant diagnostic and therapeutic challenges [5].

The pathophysiology of thrombosis in PNH is multifactorial. The absence of glycosylphosphatidylinositol (GPI)-anchored proteins, notably CD55 and CD59, on blood cells results in uncontrolled complement activation, leading to chronic intravascular hemolysis and a prothrombotic state [6]. Additional contributing factors include platelet activation, impaired fibrinolysis, and endothelial dysfunction, which together create “the most vicious acquired thrombophilic state known to medicine” [7].

The diagnosis of PNH in patients presenting with unexplained hepatic vein thrombosis is crucial, as early recognition allows for prompt initiation of targeted therapies that can dramatically improve outcomes [8]. Flow cytometry utilizing FLAER and CD55/CD59 staining remains the gold standard for confirming PNH [8].

Therapeutic management of PNH-associated BCS requires a multidisciplinary approach. Lifelong anticoagulation has traditionally been the mainstay for thrombotic events in PNH, but its efficacy is often limited due to the underlying complement-mediated pathophysiology [9]. The introduction of eculizumab, a monoclonal antibody that inhibits terminal complement component C5, has revolutionized the treatment landscape. Eculizumab not only reduces hemolysis and transfusion requirements but also markedly decreases the incidence of new thrombotic events, improving survival and quality of life [9]. Recent evidence suggests that complement inhibition therapy should be initiated promptly in PNH patients with thrombosis, as it may help maintain shunt patency in those requiring transjugular intrahepatic portosystemic shunt (TIPS) and reduce the risk of recurrent thrombosis [10].

Comparative analysis with similar published cases (Table 2) shows that early diagnosis and prompt complement inhibition improve outcomes and reduce recurrence. This reinforces the need for routine PNH screening in atypical thrombosis, particularly hepatic vein thrombosis without conventional risk factors.

**Table 2:** Comparison With Reported PNH–BCS Cases

Study	Age/Sex	PNH Clone Size	Hepatic Vein Involvement	Treatment	Outcome
Brodsky et al. (2012) [11]	25/M	95% (granulocytes)	All hepatic veins	Eculizumab + anticoagulation + TIPS	Marked improvement
Kim et al. (2017) [12]	39/M	Not specified	IVC stent thrombosis, hepatic congestion	Eculizumab	Clinical improvement
Present case	26/F	49-53% Type III	Right & middle hepatic veins	Eculizumab + anticoagulation	Marked improvement

Despite these advances, questions remain regarding the optimal duration of anticoagulation in patients receiving eculizumab, as robust data are lacking to guide cessation of anticoagulation in this setting. In select cases, allogeneic stem cell transplantation may be curative, particularly in patients with refractory disease or those who develop BCS [4].

In summary, PNH should be considered in any young patient presenting with unexplained hepatic vein thrombosis. Early diagnosis and initiation of complement inhibition therapy, alongside anticoagulation and supportive care, are critical to improving prognosis and reducing morbidity and mortality in this high-risk population.

## Conclusions

This case underscores the importance of suspecting PNH in young individuals presenting with unexplained hepatic vein thrombosis. The diagnosis was confirmed by flow cytometry, which showed a significant GPI-deficient clone consistent with classic PNH. BCS, though rare, may be the first manifestation of PNH and carries a high risk of morbidity. This highlights the crucial role of early diagnostic workup, including FLAER-based testing, in atypical presentations of thrombosis. Timely initiation of targeted therapy such as eculizumab can significantly alter the disease course, reduce thrombotic risk, and improve survival. Clinicians should maintain a high index of suspicion for PNH in similar scenarios to ensure early intervention and multidisciplinary management.
